# Supplementary figures and images for: Antitumor Activities of tRNA-Derived Fragments and tRNA Halves from Non-pathogenic Escherichia coli Strains on Colorectal Cancer and Their Structure-Activity Relationship
Source: mSystems. 2022 Apr 11;7(2):e00164-22. doi: 10.1128/msystems.00164-22 (PMC9040620; doi:10.1128/msystems.00164-22)

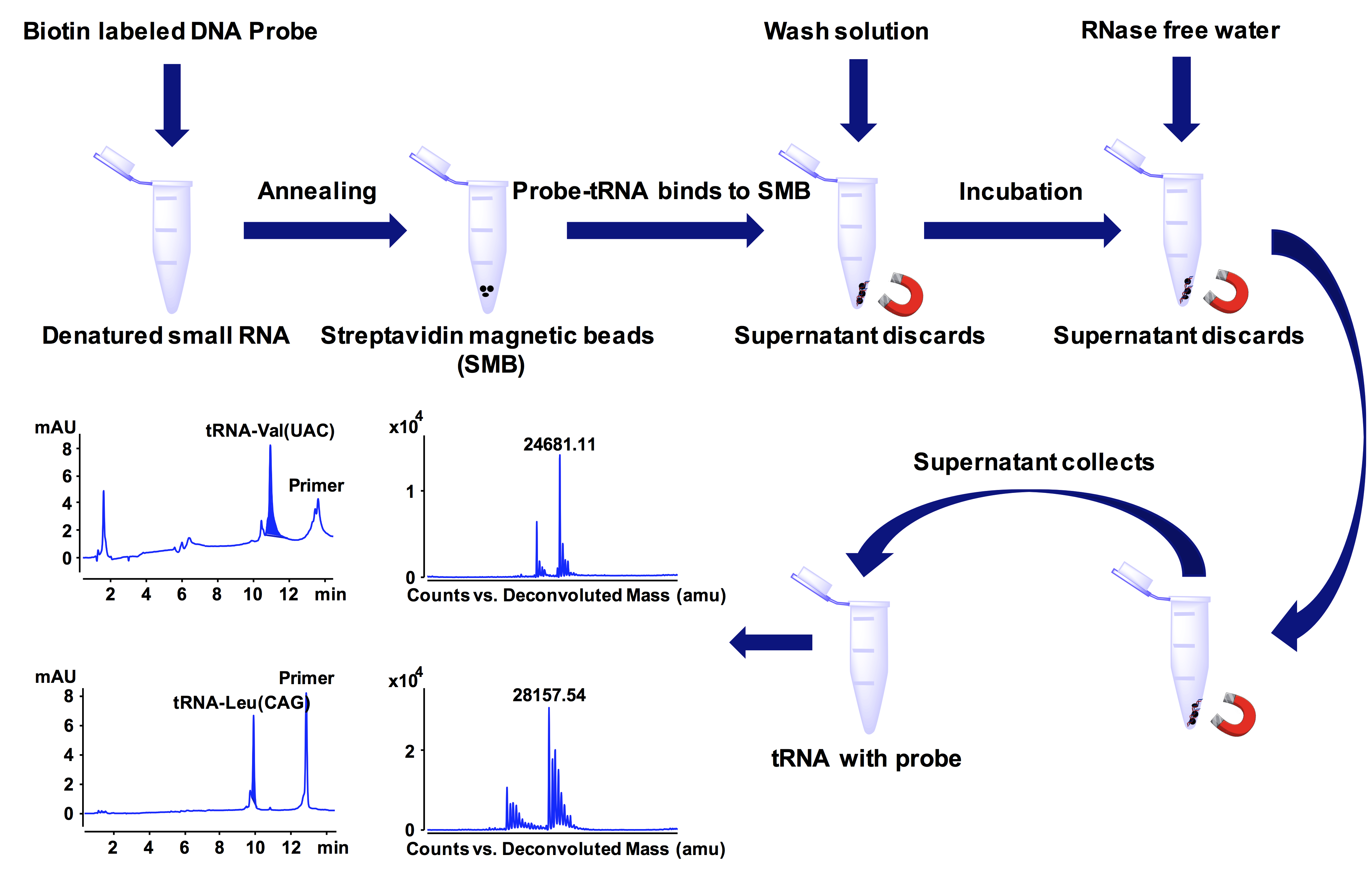

Supplement: FIG S1 [file msystems.00164-22-s0001.tif]

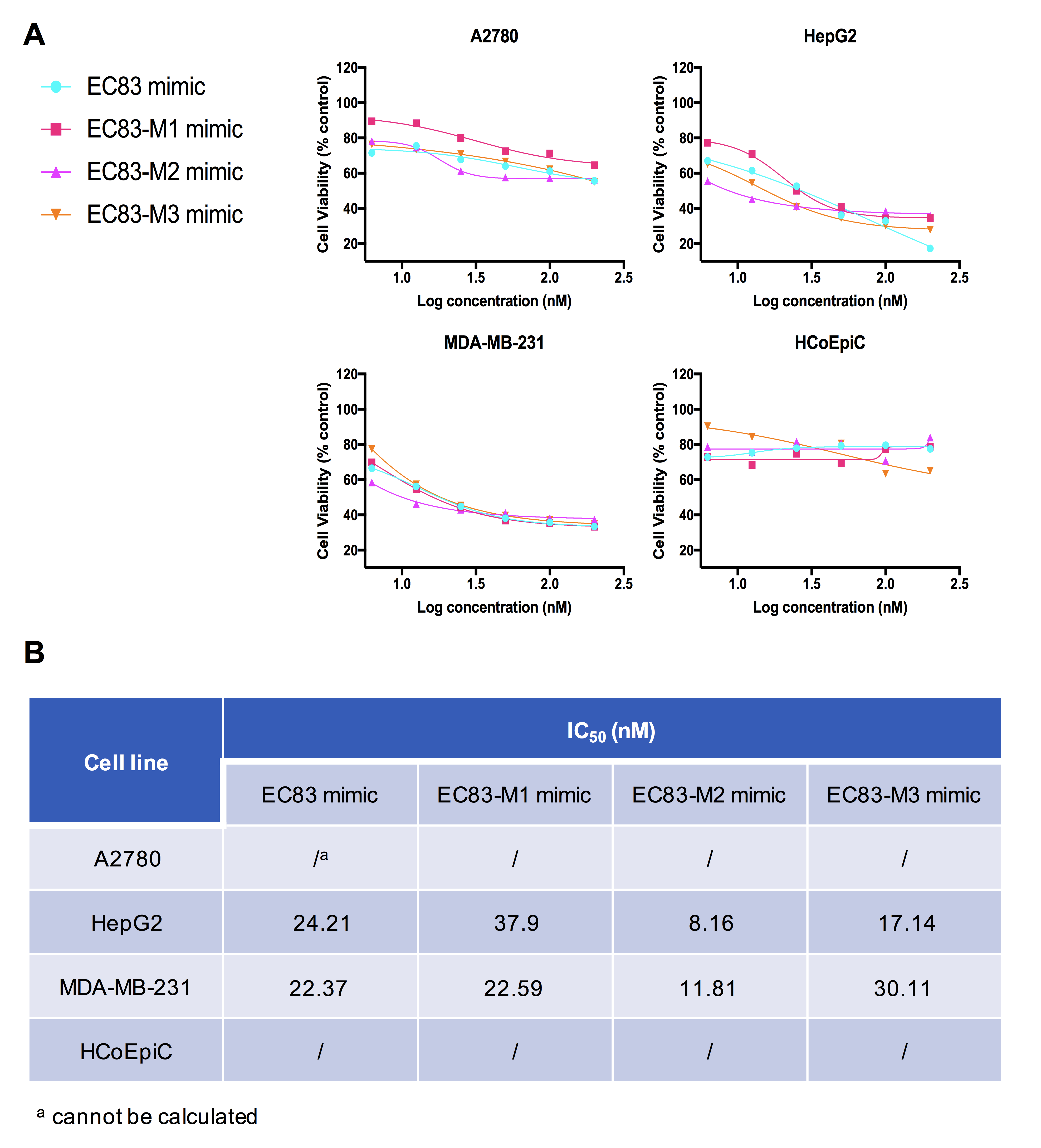

Supplement: FIG S2 [file msystems.00164-22-s0002.tif]

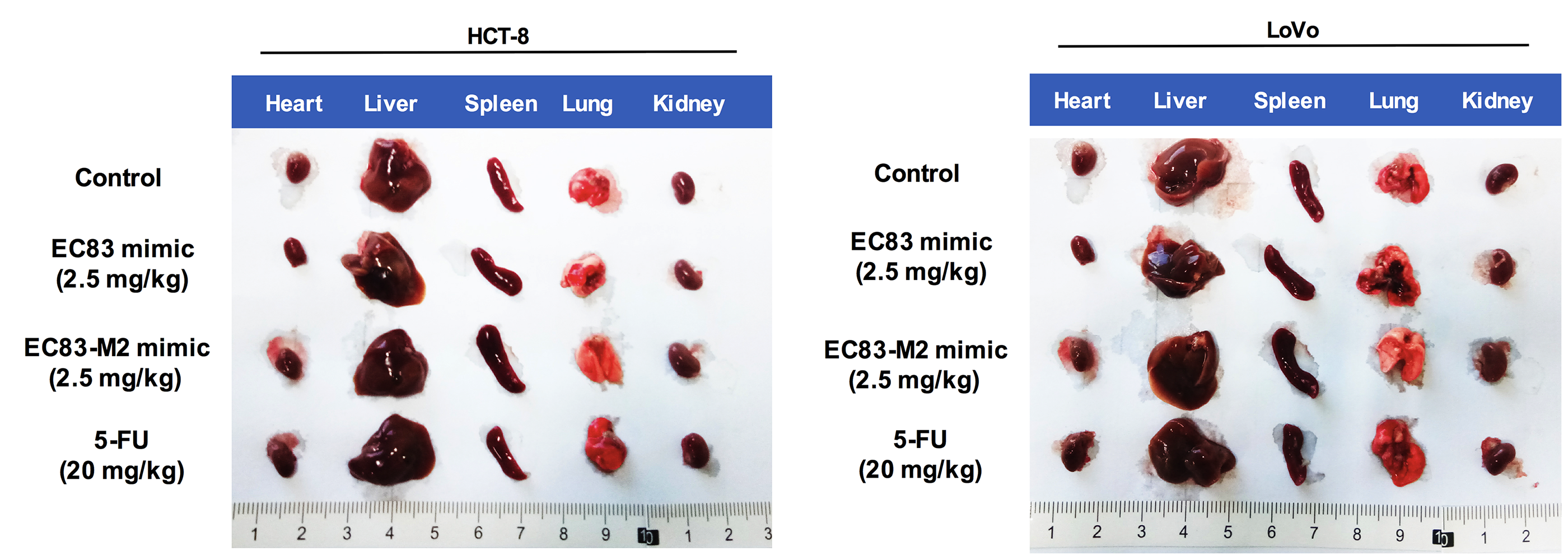

Supplement: FIG S3 [file msystems.00164-22-s0003.tif]

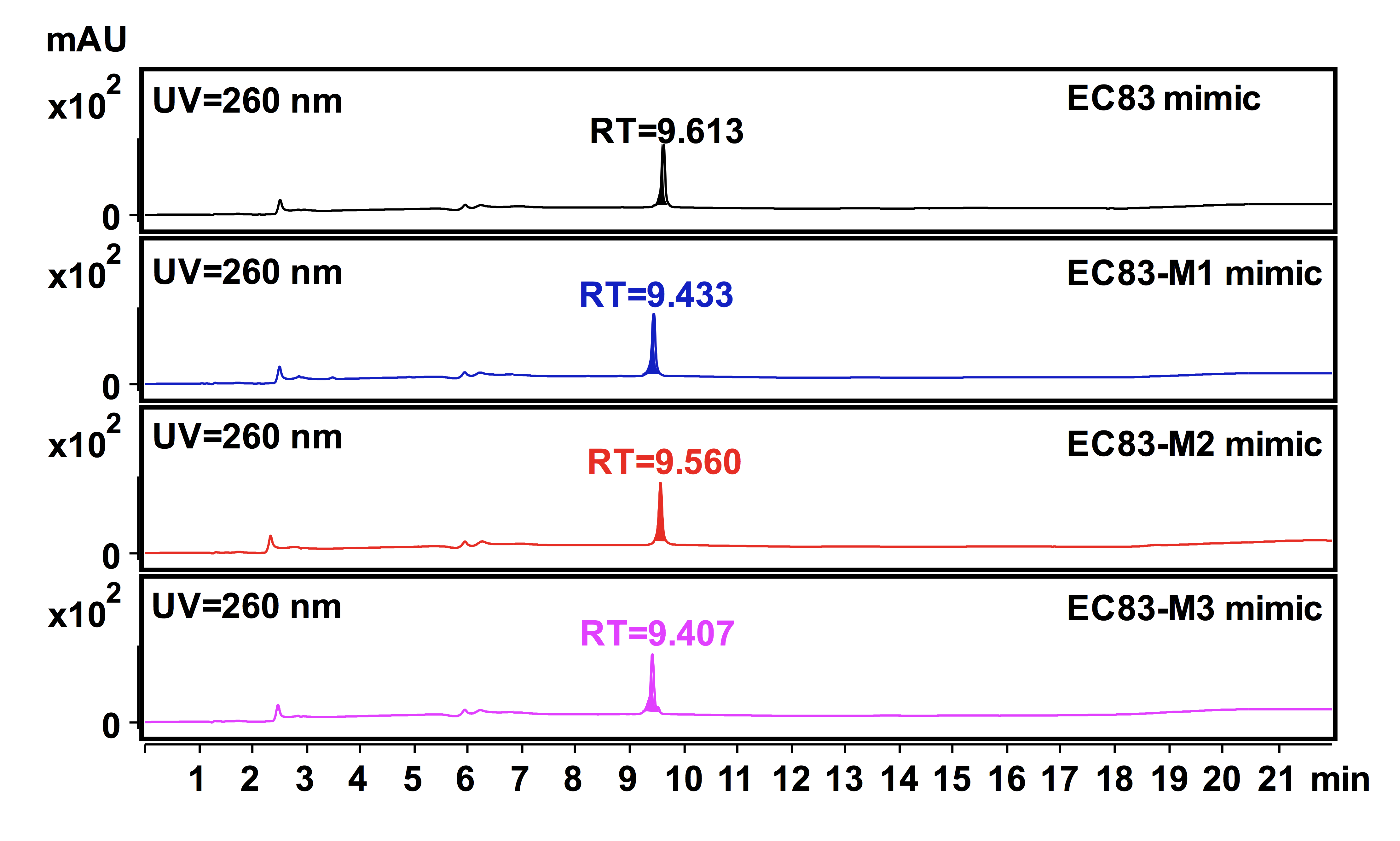

Supplement: FIG S4 [file msystems.00164-22-s0004.tif]
