# Supplementary material for: Antitumor Activities of tRNA-Derived Fragments and tRNA Halves from Non-pathogenic Escherichia coli Strains on Colorectal Cancer and Their Structure-Activity Relationship
Source: mSystems. 2022 Apr 11;7(2):e00164-22. doi: 10.1128/msystems.00164-22 (PMC9040620; doi:10.1128/msystems.00164-22)
Supplement: TABLE S1 [file msystems.00164-22-s0005.docx]

| Code | tRNA | Sequence information (5’-3’) |
| --- | --- | --- |
| 1 | tRNA-Ala(UGC) | GGGGGCA4AGCUCAGCDGGGAGAGCGCCUGCUUVGCACGCAGGAG7UCUGCGGTPCGAUCCCGCGCGCUCCCACCA |
| 2 | tRNA-Ala(UGC) | GGGGCUAUAGCUCAGCDGGGAGAGCGCCUGCUUVGCACGCAGGAG7UCUGCGGTPCGAUCCCGCAUAGCUCCACCA |
| 3 | tRNA-Ala(GGC) | GGGGCUANAGCUCAGCDGGGAGAGCGCUUGCAUGGCAUGCAAGAG7UCAGCGGTPCGAUCCCGCUUAGCUCCACCA |
| 4 | tRNA-Arg(CCG) | GCGCCCGUAGCUCAGCDGGADAGAGCGCUGCC%UCCGKAGGCAGAG7UCUCAGGTPCGAAUCCUGUCGGGCGCGCCA |
| 5 | tRNA-Arg(ICG) | GCAUCCG4AGCUCAGCDGGDAGAGUACUCGG%UICG/ACCGAGCG7XCGGAGGTPCGAAUCCUCCCGGAUGCACCA |
| 6 | tRNA-Arg(ICG) | GCAUCCG4AGCUCAGCDGGADAGAGUACUCGGCUICG/ACCGAGCG7XCGGAGGTPCGAAUCCUCCCGGAUGCACCA |
| 7 | tRNA-Arg(UCU) | GUCCUCUUAGUUAAAUGGADAUAACGAGCCC%U{CU6AGGGCUAAUUGCAGGTPCGAUUCCUGCAGGGGACACCA |
| 8 | tRNA-Arg(UCU} | GCGCCCUUAGCUCAGUUGGAUAGAGCAACGAC%U{CU6AGPCGUGGGCCGCAGGTPCGAAUCCUGCAGGGCGCGCCA |
| 9 | tRNA-Asn(GUU) | UCCUCUG[s4U]AGUUCAGDCGGDAGAACGGCGGACUQUU[t6A]AYCCGUAU[m7G]UCACUGG[m5U]YCGAGUCCAGUCAGAGGAGCCA |
| 10 | tRNA-Asp(QUC) | GGAGCGG[s4U]AGUUCAGDCGGDDAGAAUACCUGCCU[gluQ]UC[m2A]CGCAGGGG[m7G]UCGCGGG[m5U]YCGAGUCCCGYCCGUUCCGCCA |
| 11 | tRNA-Cys(GCA) | GGCGCGU[s4U]AACAAAGCGGDDAUGUAGCGGAYUGCA[ms2i6A]AYCCGUCUAGUCCGG[m5U]YCGACUCCGGAACGCGCCUCCA |
| 12 | tRNA-Gln(UUG) | UGGGGUA[s4U]CGCCAAGC[Gm]GDAAGGCACCGGU[Um]U[cmnm5s2U]UG[m2A]YACCGGCAUUCCCUGG[m5U]YCGAAUCCAGGUACCCCAGCCA |
| 13 | tRNA-Gln(CUG) | UGGGGUA[s4U]CGCCAAGC[Gm]GDAAGGCACCGGA[Um]UCUG[m2A]YYCCGGCAUUCCGAGG[m5U]YCGAAUCCUCGUACCCCAGCCA |
| 14 | tRNA-Glu(UUC) | GUCCCCUUCGUCPAGAGGCCCAGGACACCGCCCUSUC/CGGCGGUAACAGGGGTPCGAAUCCCCUGGGGGACGCCA |
| 15 | tRNA-Glu(UUC) | GUCCCCUUCGUCPAGAGGCCCAGGACACCGCCCUSUC/CGGCGGUAACAGGGGTPCGAAUCCCCUAGGGGACGCCA |
| 16 | tRNA-Glu(UUC) | GUCCCCUUCGUCPAGAGGCCAGGACACCGCCCUSUC/CGGCGGUAACAGGGGTPCGAAUCCCCUAGGGGACGCCA |
| 17 | tRNA-Gly(CCC) | GCGGGCG[s4U]AGUUCAAUGGDAGAACGAGAGCUUCCCAAGCUCUAUACGAGGG[m5U]YCGAUUCCCUUCGCCCGCUCCA |
| 18 | tRNA-Gly(UCC} | GCGGGCAUCGUAUAAUGGCUAUUACCUCAGCCU[mnm5U]CCAAGCUGAUGAUGCGGG[m5U]YCGAUUCCCGCUGCCCGCUCCA |
| 19 | tRNA-Gly(GCC) | GCGGGAAUAGCUCAGDDGGDAGAGCACGACCUUGCCAAGGUCGGG[m7G]UCGCGAG[m5U]YCGAGUCUCGUUUCCCGCUCCA |
| 20 | tRNA-His(GUG) | GGUGGCUA[s4U]AGCUCAGDDGGDAGAGCCCUGGAUUQUG[m2A]YYCCAGUU[m7G]UCGUGGG[m5U]YCGAAUCCCAUUAGCCACCCCA |
| 21 | tRNA-Ile(GAU) | AGGCUUGUAGCUCAGGDGGDDAGAGCGCACCCCUGAU[t6A]AGGGUGAG[m7G][acp3U]CGGUGG[m5U]YCAAGUCCACYCAGGCCUACCA |
| 22 | tRNA-Ile(GAU) | AGGCUUGUAGCUCAGGUGGDDAGAGCGCACCCCUGAU6AGGGUGAG7XCGGUGGTPCAAGUCCACPCAGGCCUACCA |
| 23 | tRNA-Ile(CAU) | GGCCCCU[s4U]AGCUCAGU[Gm]GDDAGAGCAGGCGACU[k2C]AU[t6A]AYCGCUUG[m7G][acp3U]CGCUGG[m5U]YCAAGUCCAGCAGGGGCCACCA |
| 24 | tRNA-Leu(CAG) | GCGAAGGUGGCGGAADD[Gm]GDAGACGCGCUAGCUUCAG[m1G]YGYUAGUGUCCUUACGGACGUGGGGG[m5U]YCAAGUCCCCCCCCUCGCACCA |
| 25 | tRNA-Leu(GAG) | GCCGAGGUGGUGGAADD[Gm]GDAGACACGCUACCUUGAG[m1G]YGGUAGUGCCCAAUAGGGCUUACGGG[m5U]YCAAGUCCCGUCCUCGGUACCA |
| 26 | tRNA-Leu(AAA) | GCCCGGA[s4U]GGUGGAADC[Gm]GDAGACACAAGGGAYU[cmnm5Um]AA[ms2i6A]AYCCCUCGGCGUUCGCGCUGUGCGGG[m5U]YCAAGUCCCGCUCCGGGUACCA |
| 27 | tRNA-Leu(CAA) | GCCGAAG[s4U]GGCGAAADC[Gm]GDAGACGCAGUUGAYU[Cm]AA[ms2i6A]AYCAACCGUAGAAAUACGUGCCGG[m5U]YCGAGUCCGGCCUUCGGCACCA |
| 28 | tRNA-Lys(UUU) | GGGUCGUUAGCUCAGDDGGDAGAGCAGUUGACU[mnm5s2U]UU[t6A]AYCAAUUG[m7G][acp3U]CGCAGG[m5U]YCGAAUCCUGCACGACCCACCA |
| 29 | tRNA-Met(CAU) | GGCUACG[s4U]AGCUCAGDD[Gm]GDDAGAGCACAUCACU[ac4C]AU[t6A]AYGAUGGG[m7G][acp3U]CACAGG[m5U]YCGAAUCCCGUCGUAGCCACCA |
| 30 | tRNA-Phe(GAA) | GCCCGGA[s4U]AGCUCAGDCGGDAGAGCAGGGGAYUGAA[ms2i6A]AYCCCCGU[m7G][acp3U]CCUUGG[m5U]YCGAUUCCGAGUCCGGGCACCA |
| 31 | tRNA-Pro(CGG) | CGGUGAUUGGCGCAGCCUGGDAGCGCACUUCGUUCGG[m1G]ACGAAGGG[m7G]UCGGAGG[m5U]YCGAAUCCUCUAUCACCGACCA |
| 32 | tRNA-Sec(UCA) | AAGAUCG[s4U]CGUCUCCGGDGAGGCGGCUGGACUUCA[i6A]AUCCAGUUGGGGCCGCGCGGUCCCGGGCAGG[m5U]YCGACUCCUGUGAUCUUGCCA |
| 33 | tRNA-Ser(UGA) | GGAAGUG[s4U]GGCCGAGC[Gm]GDDGAAGGCACCGGU[Cm]U[cmo5U]GA[ms2i6A]AACCGGCGACCCGAAAGGGUUCCAGAG[m5U]YCGAAUCUCUGCGCUUCCGCCA |
| 34 | tRNA-Ser(CGA) | GGAGAGAUGCCGGAGC[Gm]GCDGAACGGACCGGUCUCGA[ms2i6A]AACCGGAGUAGGGGCAACUCUACCGGGGG[m5U]YCAAAUCCCCCUCUCUCCGCCA |
| 35 | tRNA-Ser(GCU) | GGUGAGG[s4U]GGCCGAGAGGCDGAAGGCGCUCCC[s2C]UGCU[t6A]AGGGAGUAUGCGGUCAAAAGCUGCAUCCGGGG[m5U]YCGAAUCCCCGCCUCACCGCCA |
| 36 | tRNA-Ser(GGA) | GGUGAGG[s4U]GUCCGAGU[Gm]GDDGAAGGAGCACGCCUGGAAAGYGUGUAUACGGCAACGUAUCGGGGG[m5U]YCGAAUCCCCCCCUCACCGCCA |
| 37 | tRNA-Ser(GGA) | GGUGAGGUGUCCGAGU#GCDGAAGGAGCACGCCUGGAAAGPGUGUAUACGGCAACGUAUCGGGGGTPCGAAUCCCCCCCUCACCGCCA |
| 38 | tRNA-Thr(GGU) | GCUGAUAUGGCUCAGDDGGDAGAGCGCACCCUUGGU[m6t6A]AGGGUGAG[m7G]UCCCCAG[m5U]YCGACUCUGGGUAUCAGCACCA |
| 39 | tRNA-Thr(GGU) | GCUGAUAUAGCUCAGDDGGDAGAGCGCACCCUUGGU[m6t6A]AGGGUGAG[m7G]UCGGCAG[m5U]YCGAAUCUGCCUAUCAGCACCA |
| 40 | tRNA-Trp(CCA) | AGGGGCG[s4U]AGUUCAADDGGDAGAGCACCGGU[Cm]UCCA[ms2i6A]AACCGGGU[m7G]UUGGGAG[m5U]YCGAGUCUCUCCGCCCCUGCCA |
| 41 | tRNA-Tyr(QUA) | GGUGGGG[s4U][s4U]CCCGAGC[Gm]GCCAAAGGGAGCAGACUQUA[ms2i6A]AYCUGCCGUCAUCGACUUCGAAGG[m5U]YCGAAUCCUUCCCCCACCACCA |
| 42 | tRNA-Tyr(QUA) | GGUGGGG[s4U][s4U]CCCGAGC[Gm]GCCAAAGGGAGCAGACUQUA[ms2i6A]AYCUGCCGUCACAGACUUCGAAGG[m5U]YCGAAUCCUUCCCCCACCACCA |
| 43 | tRNA-Val(UAC) | GGGUGAU[s4U]AGCUCAGCDGGGAGAGCACCUCCCU[cmo5U]AC[m6A]AGGAGGGG[m7G]UCGGCGG[m5U]YCGAUCCCGUCAUCACCCACCA |
| 44 | tRNA-Val(GAC) | GCGUCCG[s4U]AGCUCAGDDGGDDAGAGCACCACCUUGACAUGGUGGGG[m7G][acp3U]CGGUGG[m5U]YCGAGUCCACUCGGACGCACCA |
| 45 | tRNA-Val(GAC) | GCGUUCA[s4U]AGCUCAGDDGGDDAGAGCACCACCUUGACAUGGUGGGG[m7G][acp3U]CGUUGG[m5U]YCGAGUCCAAUUGAACGCACCA |
| 46 | tRNA-Ini(CAU) | CGCGGGG[s4U]GGAGCAGCCUGGDAGCUCGUCGGG[Cm]UCAUAACCCGAAG[m7G]UCGUCGG[m5U]YCAAAUCCGGCCCCCGCAACCA |
| 47 | tRNA-Ini(CAU) | CGCGGGG[s4U]GGAGCAGCCUGGDAGCUCGUCGGG[Cm]UCAUAACCCGAAGAUCGUCGG[m5U]YCAAAUCCGGCCCCCGCAACCA |
