# Supplementary material for: Antitumor Activities of tRNA-Derived Fragments and tRNA Halves from Non-pathogenic Escherichia coli Strains on Colorectal Cancer and Their Structure-Activity Relationship
Source: mSystems. 2022 Apr 11;7(2):e00164-22. doi: 10.1128/msystems.00164-22 (PMC9040620; doi:10.1128/msystems.00164-22)
Supplement: TABLE S2 [file msystems.00164-22-s0006.docx]

| **Origin** | **Code**  **(mimic)** | **Antisense derived from tRNA**  **（5’-3’）** | **Sense**  **（5’-3’）** | **Type** |
| --- | --- | --- | --- | --- |
| tRNA-Cys(GCA) | EC1 | GGCGCGUUAACAAAGCGGUUAU | AUAACCGCUUUGUUAACGCGCC | 5'-t |
|  | EC2 | UCGACUCCGGAACGCGCCUCCA | UGGAGGCGCGUUCCGGAGUCGA | 3'-t |
| tRNA-His(GUG) | EC3 | GGUGGCUAUAGCUCAGUUGGUA | UACCAACUGAGCUAUAGCCACC | 5'-t |
|  | EC4 | UCGAAUCCCAUUAGCCACCCCA | UGGGGUGGCUAAUGGGAUUCGA | 3'-t |
| tRNA-Lys(UUU) | EC5 | GGGUCGUUAGCUCAGUUGGUAG | CUACCAACUGAGCUAACGACCC | 5'-t |
|  | EC6 | UCGAAUCCUGCACGACCCACCA | UGGUGGGUCGUGCAGGAUUCGA | 3'-t |
| tRNA-Met(CAU) | EC7 | GGCUACGUAGCUCAGUUGGUUA | UAACCAACUGAGCUACGUAGCC | 5'-t |
|  | EC8 | UCGAAUCCCGUCGUAGCCACCA | UGGUGGCUACGACGGGAUUCGA | 3'-t |
| tRNA-Asn(GUU) | EC9 | UCCUCUGUAGUUCAGUCGGUAG | CUACCGACUGAACUACAGAGGA | 5'-t |
|  | EC10 | UCGAGUCCAGUCAGAGGAGCCA | UGGCUCCUCUGACUGGACUCGA | 3'-t |
| tRNA-Phe(GAA) | EC11 | GCCCGGAUAGCUCAGUCGGUAG | CUACCGACUGAGCUAUCCGGGC | 5'-t |
|  | EC12 | UCGAUUCCGAGUCCGGGCACCA | UGGUGCCCGGACUCGGAAUCGA | 3'-t |
| tRNA-Trp(CCA) | EC13 | AGGGGCGUAGUUCAAUUGGUAG | CUACCAAUUGAACUACGCCCCU | 5'-t |
|  | EC14 | UCGAGUCUCUCCGCCCCUGCCA | UGGCAGGGGCGGAGAGACUCGA | 3'-t |
| tRNA-Asp(QUC) | EC15 | GGAGCGGUAGUUCAGUCGGUUA | UAACCGACUGAACUACCGCUCC | 5'-t |
|  | EC16 | UCGAGUCCCGUCCGUUCCGCCA | UGGCGGAACGGACGGGACUCGA | 3'-t |
| tRNA-Pro(CGG) | EC17 | CGGUGAUUGGCGCAGCCUGGUA | UACCAGGCUGCGCCAAUCACCG | 5'-t |
|  | EC18 | UCGAAUCCUCUAUCACCGACCA | UGGUCGGUGAUAGAGGAUUCGA | 3'-t |
| tRNA-Sec(UCA) | EC19 | AAGAUCGUCGUCUCCGGUGAGG | CCUCACCGGAGACGACGAUCUU | 5'-t |
|  | EC20 | UUCGACUCCUGUGAUCUUGCCA | UGGCAAGAUCACAGGAGUCGAA | 3'-t |
| tRNA-Ala(GGC) | EC21 | GGGGCUAUAGCUCAGCUGGGAG | CUCCCAGCUGAGCUAUAGCCCC | 5'-t |
|  | EC22 | UCGAUCCCGCUUAGCUCCACCA | UGGUGGAGCUAAGCGGGAUCGA | 3'-t |
| tRNA-Ala(UGC) | EC23 | GGGGGCAUAGCUCAGCUGGGAG | CUCCCAGCUGAGCUAUGCCCCC | 5'-t |
|  | EC24 | UCGAUCCCGCGCGCUCCCACCA | UGGUGGGAGCGCGCGGGAUCGA | 3'-t |
| tRNA-Ala(UGC) | EC25 | GGGGCUAUAGCUCAGCUGGGAG | CUCCCAGCUGAGCUAUAGCCCC | 5'-t |
|  | EC26 | UCGAUCCCGCAUAGCUCCACCA | UGGUGGAGCUAUGCGGGAUCGA | 3'-t |
| tRNA-Ser(CGA) | EC27 | GGAGAGAUGCCGGAGCGGCUGA | UCAGCCGCUCCGGCAUCUCUCC | 5'-t |
|  | EC28 | UCAAAUCCCCCUCUCUCCGCCA | UGGCGGAGAGAGGGGGAUUUGA | 3'-t |
| tRNA-Ser(GCU) | EC29 | GGUGAGGUGGCCGAGAGGCUGA | UCAGCCUCUCGGCCACCUCACC | 5'-t |
|  | EC30 | UCGAAUCCCCGCCUCACCGCCA | UGGCGGUGAGGCGGGGAUUCGA | 3'-t |
| tRNA-Ser(GGA) | EC31 | GGUGAGGUGUCCGAGUGGCUGA | UCAGCCACUCGGACACCUCACC | 5'-t |
|  | EC32 | UCGAAUCCCCCCCUCACCGCCA | UGGCGGUGAGGGGGGGAUUCGA | 3'-t |
| tRNA-Ser(GGA) | EC33 | GGUGAGGUGUCCGAGUGGUUGA | UCAACCACUCGGACACCUCACC | 5'-t |
|  | EC34 | UCGAAUCCCCCCCUCACCGCCA | UGGCGGUGAGGGGGGGAUUCGA | 3'-t |
| tRNA-Ser(UGA) | EC35 | GGAAGUGUGGCCGAGCGGUUGA | UCAACCGCUCGGCCACACUUCC | 5'-t |
|  | EC36 | UCGAAUCUCUGCGCUUCCGCCA | UGGCGGAAGCGCAGAGAUUCGA | 3'-t |
| tRNA-Val(GAC) | EC37 | GCGUCCGUAGCUCAGUUGGUUA | UAACCAACUGAGCUACGGACGC | 5'-t |
|  | EC38 | UCGAGUCCACUCGGACGCACCA | UGGUGCGUCCGAGUGGACUCGA | 3'-t |
| tRNA-Val(GAC) | EC39 | GCGUUCAUAGCUCAGUUGGUUA | UAACCAACUGAGCUAUGAACGC | 5'-t |
|  | EC40 | UCGAGUCCAAUUGAACGCACCA | UGGUGCGUUCAAUUGGACUCGA | 3'-t |
| tRNA-Val(UAC) | EC41 | GGGUGAUUAGCUCAGCUGGGAG | CUCCCAGCUGAGCUAAUCACCC | 5'-t |
|  | EC42 | UCGAUCCCGUCAUCACCCACCA | UGGUGGGUGAUGACGGGAUCGA | 3'-t |
| tRNA-Arg(CCG) | EC43 | GCGCCCGUAGCUCAGCUGGAUA | UAUCCAGCUGAGCUACGGGCGC | 5'-t |
|  | EC44 | UCGAAUCCUGUCGGGCGCGCCA | UGGCGCGCCCGACAGGAUUCGA | 3'-t |
| tRNA-Arg(ICG) | EC45 | GCAUCCGUAGCUCAGCUGGUAG | CUACCAGCUGAGCUACGGAUGC | 5'-t |
|  | EC46 | UCGAAUCCUCCCGGAUGCACCA | UGGUGCAUCCGGGAGGAUUCGA | 3'-t |
| tRNA-Arg(CCG) | EC47 | GCAUCCGUAGCUCAGCUGGAUA | UAUCCAGCUGAGCUACGGAUGC | 5'-t |
|  | EC48 | UCGAAUCCUCCCGGAUGCACCA | UGGUGCAUCCGGGAGGAUUCGA | 3'-t |
| tRNA-Arg(UCU) | EC49 | GUCCUCUUAGUUAAAUGGAUAU | AUAUCCAUUUAACUAAGAGGAC | 5'-t |
|  | EC50 | UCGAUUCCUGCAGGGGACACCA | UGGUGUCCCCUGCAGGAAUCGA | 3'-t |
| tRNA-Arg(UCU) | EC51 | GCGCCCUUAGCUCAGUUGGAUA | UAUCCAACUGAGCUAAGGGCGC | 5'-t |
|  | EC52 | UCGAAUCCUGCAGGGCGCGCCA | UGGCGCGCCCUGCAGGAUUCGA | 3'-t |
| tRNA-Gln(CUG) | EC53 | UGGGGUAUCGCCAAGCGGUAAG | CUUACCGCUUGGCGAUACCCCA | 5'-t |
|  | EC54 | UCGAAUCCUCGUACCCCAGCCA | UGGCUGGGGUACGAGGAUUCGA | 3'-t |
| tRNA-Gln(UUG) | EC55 | UGGGGUAUCGCCAAGCGGUAAG | CUUACCGCUUGGCGAUACCCCA | 5'-t |
|  | EC56 | UCGAAUCCAGGUACCCCAGCCA | UGGCUGGGGUACCUGGAUUCGA | 3'-t |
| tRNA-Ile(GAU) | EC57 | AGGCUUGUAGCUCAGGUGGUUA | UAACCACCUGAGCUACAAGCCU | 5'-t |
|  | EC58 | UCAAGUCCACUCAGGCCUACCA | UGGUAGGCCUGAGUGGACUUGA | 3'-t |
| tRNA-Ile(GAU) | EC59 | AGGCUUGUAGCUCAGGUGGUUA | UAACCACCUGAGCUACAAGCCU | 5'-t |
|  | EC60 | UCAAGUCCACUCAGGCCUACCA | UGGUAGGCCUGAGUGGACUUGA | 3'-t |
| tRNA-Ile(UAU) | EC61 | GGCCCCUUAGCUCAGUGGUUAG | CUAACCACUGAGCUAAGGGGCC | 5'-t |
|  | EC62 | UCAAGUCCAGCAGGGGCCACCA | UGGUGGCCCCUGCUGGACUUGA | 3'-t |
| tRNA-Thr(GGU) | EC63 | GCUGAUAUAGCUCAGUUGGUAG | CUACCAACUGAGCUAUAUCAGC | 5'-t |
|  | EC64 | UCGAAUCUGCCUAUCAGCACCA | UGGUGCUGAUAGGCAGAUUCGA | 3'-t |
| tRNA-Thr(GGU) | EC65 | GCUGAUAUGGCUCAGUUGGUAG | CUACCAACUGAGCCAUAUCAGC | 5'-t |
|  | EC66 | UCGACUCUGGGUAUCAGCACCA | UGGUGCUGAUACCCAGAGUCGA | 3'-t |
| tRNA-Glu(UUC) | EC67 | GUCCCCUUCGUCUAGAGGCCCA | UGGGCCUCUAGACGAAGGGGAC | 5'-t |
|  | EC68 | UCGAAUCCCCUGGGGGACGCCA | UGGCGUCCCCCAGGGGAUUCGA | 3'-t |
| tRNA-Glu(UUC) | EC69 | GUCCCCUUCGUCUAGAGGCCCA | UGGGCCUCUAGACGAAGGGGAC | 5'-t |
|  | EC70 | UCGAAUCCCCUAGGGGACGCCA | UGGCGUCCCCUAGGGGAUUCGA | 3'-t |
| tRNA-Glu(UUC) | EC71 | GUCCCCUUCGUCUAGAGGCCAG | CUGGCCUCUAGACGAAGGGGAC | 5'-t |
|  | EC72 | UCGAAUCCCCUAGGGGACGCCA | UGGCGUCCCCUAGGGGAUUCGA | 3'-t |
| tRNA-lni(CAU) | EC73 | CGCGGGGUGGAGCAGCCUGGUA | UACCAGGCUGCUCCACCCCGCG | 5'-t |
|  | EC74 | UCAAAUCCGGCCCCCGCAACCA | UGGUUGCGGGGGCCGGAUUUGA | 3'-t |
| tRNA-lni(CAU) | EC75 | CGCGGGGUGGAGCAGCCUGGUA | UACCAGGCUGCUCCACCCCGCG | 5'-t |
|  | EC76 | UCAAAUCCGGCCCCCGCAACCA | UGGUUGCGGGGGCCGGAUUUGA | 3'-t |
| tRNA-Gly(CCC) | EC77 | GCGGGCGUAGUUCAAUGGUAGA | UCUACCAUUGAACUACGCCCGC | 5'-t |
|  | EC78 | UCGAUUCCCUUCGCCCGCUCCA | UGGAGCGGGCGAAGGGAAUCGA | 3'-t |
| tRNA-Gly(GCC) | EC79 | GCGGGAAUAGCUCAGUUGGUAG | CUACCAACUGAGCUAUUCCCGC | 5'-t |
|  | EC80 | UCGAGUCUCGUUUCCCGCUCCA | UGGAGCGGGAAACGAGACUCGA | 3'-t |
| tRNA-Gly(UCC) | EC81 | GCGGGCAUCGUAUAAUGGCUAU | AUAGCCAUUAUACGAUGCCCGC | 5'-t |
|  | EC82 | UCGAUUCCCGCUGCCCGCUCCA | UGGAGCGGGCAGCGGGAAUCGA | 3'-t |
| tRNA-Leu(CAA) | EC83 | GCCGAAGUGGCGAAAUCGGUAG | CUACCGAUUUCGCCACUUCGGC | 5'-t |
|  | EC84 | UCGAGUCCGGCCUUCGGCACCA | UGGUGCCGAAGGCCGGACUCGA | 3'-t |
| tRNA-Leu(CAG) | EC85 | GCGAAGGUGGCGGAAUUGGUAG | CUACCAAUUCCGCCACCUUCGC | 5'-t |
|  | EC86 | UCAAGUCCCCCCCCUCGCACCA | UGGUGCGAGGGGGGGGACUUGA | 3'-t |
| tRNA-Leu(GAG) | EC87 | GCCGAGGUGGUGGAAUUGGGAG | CUCCCAAUUCCACCACCUCGGC | 5'-t |
|  | EC88 | UCAAGUCCCGUCCUCGGUACCA | UGGUACCGAGGACGGGACUUGA | 3'-t |
| tRNA-Leu(AAA) | EC89 | GCCCGGAUGGUGGAAUCGGUAG | CUACCGAUUCCACCAUCCGGGC | 5'-t |
|  | EC90 | UCAAGUCCCGCUCCGGGUACCA | UGGUACCCGGAGCGGGACUUGA | 3'-t |
| tRNA-Tyr(QUA) | EC91 | GGUGGGGUUCCCGAGCGGCCAA | UUGGCCGCUCGGGAACCCCACC | 5'-t |
|  | EC92 | UCGAAUCCUUCCCCCACCACCA | UGGUGGUGGGGGAAGGAUUCGA | 3'-t |
| tRNA-Tyr(QUA) | EC93 | GGUGGGGUUCCCGAGCGGCCAA | UUGGCCGCUCGGGAACCCCACC | 5'-t |
|  | EC94 | UCGAAUCCUUCCCCCACCACCA | UGGUGGUGGGGGAAGGAUUCGA | 3'-t |
